# Dr. Barlow

**Published:** 1844-07

**Authors:** 


### OBITUARY—Dr. Barlow.

[For the following brief memoir of Dr. Barlow's life and writings we are indebted to the pages of the Provincial Medical and Surgical Journal. Although written (as there stated) by "one of Dr. Barlow's intimate friends," (Mr. Soden,) it is far from over-colouring the character or over-stating the merits of that excellent man and accomplished physician. We speak from long personal knowledge of his worth and we say deliberately, we have never known a man possessed of more gentlemanly and honorable feelings, or of a more truly philanthropic, liberal, and patriotic spirit than our lamented friend.]

Edward Barlow was born at Mullingar, county of Meath, Ireland, on the 25th of June, 1779. His father was a distinguished practitioner at that place, and died August 9, 1825, at the advanced age of eighty-one. The Westmeath Journal, in announcing his death, stated that "he was for fifty-six years surgeon to the County Infirmary, and for the greater part of that period, until years and infirmity limited his exertions, in possession of the almost undivided medical practice of the surrounding country—a sure proof of the zeal and ability with which he exercised the arduous duties of his professional calling."

Dr. Barlow commenced his professional education under the guidance and direction of his father, who, from an early period, intended to bring up his son to the medical profession, in the expectation that he would succeed to his private practice, and public appointment at the Infirmary. In furtherance of these objects, and to enable him to become a Licentiate of the Dublin College of Surgeons, he was bound apprentice to his father, and after the expiration of his apprenticeship, attended the various lectures and hospitals in Dublin, Edinburgh, and London; at the latter place he was a pupil of Cline and Cooper, at the Borough Hospitals. He became a Licentiate of the Dublin College of Surgeons in May, 1801, and graduated at Edinburgh in June, 1803.

He resided in Dublin, and practised as a surgeon till 1807, when he came to Bath, and commenced that career as a physician, which soon led to celebrity and distinction. He cultivated medical science with zeal and assiduity, and communicated to the public the result of his reflections in various writings. He became physician successively to the Bath City Infirmary, (previously to its junction with the Casualty Hospital, when the two charities formed the United Hospital), and to the General Hospital. He performed the duties of physician to both these Hospitals in the most assiduous and laborious way. He was scarcely ever absent on his allotted days of attendance, and generally visited both Hospitals daily. At the United Hospital he usually spent from three to four hours in investigating and prescribing for the complaints of the out-door applicants, and the patience with which he listened to their histories, and the kindness of his demeanour towards them, made him a great favorite with this class. His exertions, however, were not limited to objects connected with his profession. He was the zealous and eloquent advocate of every project that was calculated to extend knowledge, promote humanity, or in any way to be useful to his fellow-creatures. He was one of the original founders of the Literary and Philosophical Institution, and ever afterwards an active member of its Committee. He was chiefly instrumental in establishing a Phrenological Society in Bath, and was always ready to furnish a paper at their meetings, when no other contributor could be found. He was a zealous supporter of the Humane Society. Amongst the poor who applied as out-patients at the United Hospital he often found that great misery arose from want of proper clothing, and to remedy in some degree this evil, he formed a charity for the purpose of distributing flannel waistcoats to such objects as appeared likely to be benefited by them. This charity is now attached to the Hospital, and has been productive of great comfort to many poor persons who were suffering from rheumatism.

Indeed, it is not saying too much in praise of Dr. Barlow, to assert that his benevolent feelings led him to support with his pen, his purse, and his personal exertions, every object of public charity and utility.

It is scarcely necessary to remind the readers of this journal how warmly he was interested in the prosperity of the Provincial Medical Association. We have all admired his zeal, and been delighted with the eloquence with which, at our annual meetings, he always advocated every measure that was likely to promote the welfare of the society, or advance the honour and respectability of the profession. At our future assemblies his absence will indeed be deplored, and we shall all feel that we have sustained a loss which cannot be easily supplied.

Dr. Barlow's private practice was considerable as a consulting physician, but not so extensive in regard to general practice as might have been expected from his talents and his zeal, united as they were with the advantage of being connected with two hospitals, both of which afforded ample experience, and presented favorable opportunities of observing disease. There can be no doubt, however, that if he had desired more extensive private employment he might have obtained it; but he loved the science and disliked the trade of medicine. He was sufficiently affluent to be satisfied with a limited income from his professional exertions, and a belief generally prevailed, that professional emolument was a matter of indifference, or, at least, of secondary importance, to him.

In all the social and domestic relations of life he was most exemplary. He was a kind husband, an affectionate father, and a sincere friend. His manners were at times reserved, but he was generous and benevolent. His habits were retired, and he was not fond of much society, but no one could receive his friends with greater hospitality and courtesy. He had a deep sense of religion, which he manifested, not so much by the observance of austere forms, as by a conscientious discharge of all the duties of life. His health began to decline about two years before his death, when he experienced a severe domestic calamity, from the shock of which he never recovered. His changed appearance at the meeting of the Association at Leeds was observed by all his friends, and from that time he progressively declined. Latterly, his emaciation became extreme, and he finally sank under the effects of disease on the 2d of April in this year.

The governors of the Bath Hospital met on the 1st of May, to elect a successor to Dr. Barlow, and before they separated the following resolution was unanimously passed, the whole body of governors simultaneously rising, as a mark of respect to the memory of one who was so universally and deservedly esteemed: "That this court desire to record the expression of their unfeigned sorrow at the decease of the late senior physician, the lamented Dr. Barlow, and their gratitude for the services which, during the period of twenty-five years, he so cheerfully and efficiently rendered. Whether they regard his skill, his humanity, his attention to the sick and suffering, or his conduct as a member of the honorable profession to which he belonged, they feel that his departure has left a blank which will not easily be filled."

Dr. Barlow has been long known to the medical profession by the extent and value of his writings, which are characterized by elegance of composition and copiousness of illustration. He wrote with great fluency and remarkable rapidity; such indeed was his ready command of language that he appeared to have the power of exhausting every subject on which he treated, almost without an effort. This facility of expressing his ideas led him probably to an early as well as a frequent use of his pen, for his first contribution to medical literature was written while he was a student. It is impossible to enumerate all Dr. Barlow's publications, as, besides his more important works, he wrote in newspapers, and other periodicals, on various topics of local and temporary interest; but the following list contains his chief medical productions:

'History of a considerable wound of the Brain, attended with singular circumstances, by Mr. Edward Barlow, student of Medicine at Edinburgh, from Westmeath, Ireland.' (Duncan's Annals of Medicine for 1802.)

'Dissertatio Medica Inauguralis, de Peritonitide Puerperarum.' Edinburgh, 1803.

'Case of Dysphagia, together with some other unusual affections, supervening on inflammation of the lungs, wherein a gum-elastic tube was advantageously employed as a passage to the stomach.' (Dublin Medical and Physical Essays, for June, 1807.)

'Observations on Medical Reform; illustrating the present condition of medical science, education, and practice throughout Great Britain and Ireland, and proposing such alterations therein, as appear most likely to succeed in remedying the several evils which abound in this profession, and which have at length become subjects of universal complaint.' Dublin, 1807.

Dr. Barlow's other works on Medical Reform were

'An attempt to develope the Fundamental Principles, which should guide the Legislature in regulating the Profession of Physic.' (Edinburgh Medical and Surgical Journal, vol. xiv.)

'Exposition of the present state of the Profession of Physic in England, and of the Laws enacted for its Government.' (Edinburgh Medical and Surgical Journal, vol. xvi.)

'An Essay on the Medical Profession, showing its natural unity, and suggesting such arrangements as would render its condition conformable to just principles of Political Science, and conducive to the interests both of the profession and the public.' (Edinburgh Medical and Surgical Journal. vol. xxviii.)

'An Essay on Medical Reform.' (London Medical Gazette, vol. xiii.)

Besides these articles, Dr. Barlow wrote the Reports that were presented to the Provincial Association on Medical Reform.

Dr. Barlow's other contributions to the Medical and Surgical Journals were:

'Pathological and Practical observations,' (Edinburgh Journal) occupying part of seven numbers in vols. ix and x.

'Case of laceration of the fibres of the gastrocnemius muscle, treated without rest or confinement.' (Ib. vol. xix.)

'A case of bronchocele successfully treated by iodine.' (Ib. vol. xxi.)

'A case in which chalybeate pills were retained for an unusual time in the intestines.' (Lancet, March, 1827.)

'An account of a tape worm.' (Midland Reporter, No. 13.)

'On spinal weakness, and some effects of incipient curvature.' (No. 14.)

'On dropsy, with coagulable urine.' (Ib. No. 16.)

The Transactions of the Provincial Medical and Surgical Association are enriched by the following productions of his pen:

'The objects and modes of medical investigation.'

'Biographical memoirs of the late Dr. Thackeray, of Bedford.' (Vol. i.)

'The retrospective address delivered at the first anniversary of the Association, held at Bristol in the year 1833.' (Vol. ii.)

'Records of ovarian tumours.' (Vol. iv.)

'Address at the meeting of the Association, at Bath.' (Vol. vii.)

'Address on vacating the chair, at Liverpool. (Vol. viii.)

He wrote the following articles in the 'Cyclopædia of Practical Medicine:'

'Antiphlogistic regimen; Congestion of blood; Determination of blood; Physical education; Gastrodynia; Gout; Plethora; and Rheumatism.

The 'Essay on Physical Education,' has been translated into most of the continental languages, and is generally admired.

In 1822 he published 'An Essay on the Medicinal Efficacy and Employment of the Bath Waters, illustrated by Remarks on the Physiology and Pathology of the Animal Frame, with reference to the Treatment of Gout, Rheumatism, Palsy, and Eruptive Diseases.'

It will be perceived that the subject of medical reform occupied Dr. Barlow's attention at an early period of his life The pamphlet published at Dublin in 1807 was a reprint of letters previously inserted in a newspaper. It was dedicated to Lord Henry Petty, the present Marquis of Lansdowne. The author did not attach his name to it; but in his preface says that "he is veiled in an obscurity which curiosity shall in vain attempt to penetrate." It may not therefore be generally known that this work was written by Dr. Barlow.

Medical reform was a favorite theme with Dr. Barlow throughout his life; and how zealously and ably he vindicated its cause must be in the recollection of most of the members of the Provincial Medical Association. He was examined before the Parliamentary committee appointed in 1834, to inquire into the state of the profession; but his evidence was never printed, having been lost in the fire that destroyed the Houses of Parliament.

I ought to have included in the list of his works one which, though not strictly medical, is on a subject sufficiently connected with our profession to be deemed interesting to and worthy of consideration by medical men. I allude to a pamphlet which he published in 1825 (anonymously), and entitled 'An Apology for the Study of Phrenology,' in which the author maintains its truth and asserts its utility. Dr. Barlow became a convert to this science at the amiable and lamented Spurzheim's first visit to Bath in 1814, and was ever afterwards its zealous advocate. He was a regular attendant at the meetings of the Phrenological Society during its existence, and read three papers on phrenological subjects before the members in 1840 and 1841.

The tribute which I now offer to the memory of our departed friend is very inadequate to his merits, but it is written by one who was long and intimately acquainted with him; one who admired his talents, esteemed his virtues, and will ever sincerely lament his death. It was not my intention to give a complete history of his life; and if this brief sketch of his character should disappoint the expectations of his friends, they may derive consolation from the assurance that his works have secured him a lasting fame with his profession, that his memory will be held in grateful and honorable remembrance by his numerous friends in that Association of which he was not only one of the founders, but one of its most useful, active, and eloquent members.

---

